# Repackaging and Performance Analysis of Implantable Pressure Sensor

**DOI:** 10.3390/s25030651

**Published:** 2025-01-22

**Authors:** Liu Cui, Shuangkui Wang, Kai Zhao, Zhisen Si

**Affiliations:** 1 Department of Computer Science and Information Engineering, Shanghai Institute of Technology, Shanghai 201418, China; cui_liu@foxmail.com (L.C.); 226141110@mail.sit.edu.cn (S.W.); 226142145@mail.sit.edu.cn (Z.S.); 2 School of Electronics, Information and Electrical Engineering, Shanghai Jiao Tong University, Shanghai 200240, China

**Keywords:** implantable pressure sensor, sensor repackaging, PDMS, parylene coating, electronic capsule system

## Abstract

In recent years, repackaging technology has been widely used in miniaturized implantable pressure sensors. However, the current packaging structure still has significant problems regarding biocompatibility, environmental adaptability, and measurement accuracy, which greatly limits its application in vivo measurement systems. In this paper, we report a method for implantable pressure sensor repackaging based on silicone oil, polydimethylsiloxane (PDMS) film, and polymer (parylene) coating. A systematic investigation using finite element analysis is conducted to assess the impact of packaging components on sensor performance, providing a solid theoretical foundation for packaging optimization. Experimental results demonstrate that when the parylene coating thickness is below 30 µm, the sensors exhibit superior linearity, repeatability, and reliability, along with exceptional stability and dynamic response across clinically relevant pressure ranges. This research provides valuable insights into the packaging design of implantable pressure sensors, facilitating the development of more stable, reliable, and cost-effective sensors for in vivo measurement systems.

## 1. Introduction

Changes in contemporary lifestyles and dietary habits have led to a rise in gastrointestinal (GI) disorders, making them a significant global health concern. According to the Rome IV criteria, constipation affects 40% of the population, severely impacting both health and quality of life [1]. It has been found that GI disorders are often associated with changes in GI pressure, which is typically measured using catheter manometry. While this method provides relatively accurate pressure readings, it is invasive, causes discomfort, and poses safety risks. Additionally, doctors can only make objective judgments based on partial data in abnormal physiological states [2].

With the continuous advancement of microelectromechanical system (MEMS) technology and robotics, diagnostic and treatment systems are moving toward miniaturization and intelligence. The application of implantable pressure sensors provides a new opportunity for this trend, and the diagnosis and treatment systems developed by them promises to transform traditional medical treatment which relies on single measurements into prophylactic treatment strategies through real-time continuous monitoring with high accuracy and precision [3,4]. Among these sensors, the piezoresistive pressure sensor plays a key role in monitoring vital physiological pressures in organs such as the heart, brain, eyes, and gastrointestinal tract, thanks to their compact, lightweight design and low energy consumption. These physiological pressures are not only key indicators for assessing the health status of patients but also of vital importance for medical personnel to diagnose diseases, formulate personalized treatment plans, and track the status of patients [2].

In electronic capsule systems, the traditional encapsulation process for pressure sensor modules includes critical steps such as medical-grade silicone sealing, adhesive fixation, gold wire bonding, and fine-wire soldering. While these techniques are widely used, they often introduce residual and thermal stresses during the manufacturing process. These stresses, generated from factors such as adhesive curing and PCB sealing, can lead to deformation of the sensor housing, uneven stress distribution, and eventual degradation of the sensor’s performance. This can result in reduced sensitivity, baseline drift, and overall inaccuracies in pressure measurements. While researchers have attempted to mitigate these challenges by refining soldering techniques [5], developing innovative encapsulation methods [6,7,8,9], and advancing bonding techniques [10], these improvements have only partially addressed the issues. Specifically, these sensors are required to function in complex and demanding environments, such as the human body, where conditions such as moisture infiltration, corrosion, and mechanical stress can significantly affect measurement accuracy and long-term reliability. The current optimization strategies, although beneficial, remain insufficient to fully address the stringent requirements of specialized applications, particularly in long-term implantation scenarios, where the sensors are exposed to dynamic, high-moisture environments. Thus, further innovation in sensor packaging is needed to meet the performance and longevity standards required for precise, continuous GI pressure monitoring.

To address the challenges of sensor encapsulation, a series of novel encapsulation methods have been reported, including coating encapsulation protection and filler encapsulation protection. Essentially, they use barrier materials to hermetically seal the sensor from the environment. In the study of coating repackaging structures, common materials, such as oxides, nitrides, and titanium, as well as custom hybrid coatings, have been widely used [11,12,13]. However, these materials often involve high fabrication costs and have limited adaptability across different applications. In response, attention has shifted towards biocompatible polymers, like parylene and PDMS [14,15,16], which offer excellent mechanical strength, chemical resistance, and biocompatibility [17,18]. In studies of filler repackaging structures, medical-grade silicone oil or gel is often used as the filling material, complemented by an external thin film to protect pressure sensors [19,20,21,22,23]. This approach effectively isolates the sensor from environmental disturbances while enhancing its durability and reliability. The study presented in [24], for example, examines the impact of packaging on sensor performance, particularly for sensors used in challenging environments such as the gastrointestinal tract. In their study, the authors examined various packaging methods for gas sensors with a focus on improving sensitivity and long-term stability under harsh conditions, which is directly related to the packaging of the pressure sensor packaging in our study, but only briefly analyzed specific packaging components. Although novel re-encapsulation techniques have been widely adopted [8,25,26], previous studies have focused on the use of encapsulation materials with different thicknesses, focusing mainly on individual aspects of sensing encapsulation. Therefore, a systematic study of the effect of encapsulation parameters on sensor performance is necessary.

To address the drift issue commonly observed in pressure sensor repackaging in electronic capsule systems, this paper proposes an innovative repackaging method based on a multi-layer structure of silicone oil, PDMS, and parylene. In this method, the pressure sensor is embedded in a sealed chamber filled with silicone oil, with an external barrier of PDMS and parylene layers providing effective environmental isolation. Building on this structure, we further investigate and optimize the performance of sensors with this repackaging design by systematically analyzing the influence of encapsulation parameters on sensor performance through finite element analysis. In this process, we aim to strike an optimal balance between biocompatibility and mechanical strength to ensure sensor stability while enhancing reliability and providing ideas for package design of implantable sensors.

The remainder of this paper is organized as follows: Section 2 offers a comprehensive overview of the structural design of the sensor, the model methodology employed and test platform. Section 3 prominently presents the simulation and experimental results, followed by a thorough discussion of these findings. Finally, Section 4 provides a concise conclusion to the work presented.

## 2. Materials and Methods

**Figure 1:**
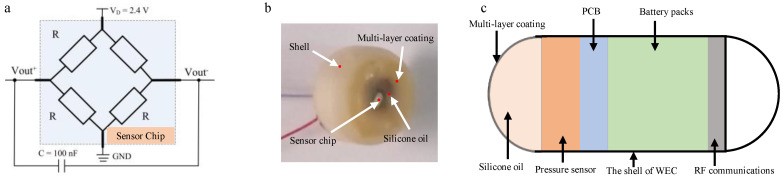
The schematic diagram of pressure detection and diagnosis. (**a**) Pressure sensor chip and related circuits. (**b**) Pressure sensor structure. (**c**) Diagnostic electronic capsule system.

As shown in Figure 1, the pressure sensor is the core component for detecting gastrointestinal pressure signals and forms the basis for analyzing gastrointestinal motility. In this study, we utilize the C29 media-resistant absolute pressure sensor chip from AKTIV SENSOR, encapsulated in an implantable sensor package made from silicone oil, polydimethylsiloxane (PDMS), and parylene coating. Parylene, used as the secondary encapsulation material, offers excellent biocompatibility and mechanical properties, effectively addressing sensor damage and failure issues. Its outstanding flexibility and ductility enable accurate transmission of even the smallest pressures, facilitating better interaction with the external environment.

At the same time, silicone oil provides additional protection for the sensor, effectively isolating it from external environmental influences and mitigating corrosion caused by factors such as water environments, reference cavity pressure, and biological molecules or cells. This dual-protection strategy not only ensures the sensor’s long-term stability but also resolves issues related to sensitivity and baseline drift. Moreover, it offers a reliable solution for the application of implantable sensors. The final design is integrated into a diagnostic electronic capsule system.

### 2.1. Structure of Pressure Sensor

**Figure 2:**
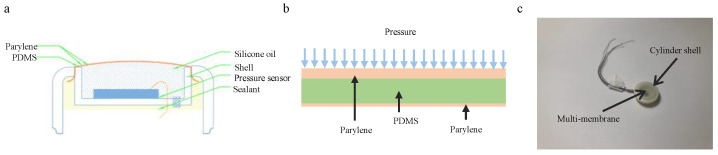
Pressure sensor packaging structure. (**a**) Pressure sensor profile. (**b**) Multi-coating structures of parylene and PDMS. (**c**) Pressure sensor physical diagram.

As shown in Figure 2, the pressure sensor is housed in a smooth, cylindrical made of acrylonitrile-butadiene-styrene (ABS) material casing with no sharp edges, and the top features an opening. The external profile of the pressure chamber is cylindrical with a concave center, forming an open container. A PDMS membrane is first coated with a dense layer of parylene to isolate the different substances on either side of the PDMS membrane. The PDMS membrane is then cut according to the size of the opening in the pressure chamber to prepare it for assembly.

The bare pressure sensor die is embedded into a PCB adapter, with the two components fixed in place using an adhesive. Bonding wires are used to connect the sensor pads on the die to the pads on the PCB adapter. The connecting wires are soldered onto the PCB and extend through the pressure chamber, connecting to the main control circuit inside the electronic capsule.

The interior of the pressure chamber is filled with interface fluid, and the cut PDMS membrane is placed over the pressure chamber. The outer casing structure is then sealed from top to bottom, enclosing both the pressure chamber and the PDMS membrane, thus forming a sealed cavity. If the initial pressure inside the sealed cavity is insufficient, a syringe can be used to puncture the rubber seal and inject additional interface fluid, thereby adjusting the internal pressure to the desired level.

### 2.2. Mathematical Model of Pressure Sensor

The performance of pressure sensors, especially those integrated into electronic capsule systems, is heavily influenced by the mechanical properties of the materials and interactions between the forces acting on the system. A comprehensive understanding of these interactions is essential for ensuring the accurate and reliable measurement of pressure. In this section, we present a mathematical model that describes the pressure sensor’s response to external pressure, focusing on the effects of surface tension, material deformation, and the stress–strain relationship.

The Young–Laplace equation is a fundamental tool for modeling the pressure difference across a curved membrane influenced by surface tension. This equation is critical for understanding how pressure is distributed across the sensor’s membrane, directly impacting its deformation and overall performance. The equation is expressed as

(1) $$\Delta P=\gamma (\frac{1}{R_{1}}+\frac{1}{R_{2}}).$$

where ΔP is the pressure difference between the interfaces, γ is the surface tension coefficient, $R_{1}$ and $R_{2}$ are the principal radius of curvature.

This equation describes how external pressure induces curvature in the membrane and dictates the way pressure is transferred within the sensor system. Understanding this relationship is crucial for optimizing the sensor design and predicting its behavior under varying external conditions.

In addition to the pressure distribution, the deformation of the sensor material under applied pressure is another critical factor affecting its performance. The relationship between the induced stress and strain in the material is governed by Hooke’s law:

(2) σ=E×ε.

where σ is the stress in the coating, *E* is the elastic modulus of the coating material, and ε is the strain within the coating.

This equation provides insights into how much the material will deform in response to a given stress, allowing us to predict the sensor’s behavior under varying load conditions. The ability to model these mechanical behaviors is essential for assessing sensor performance in real-world applications.

Together, these two models form a robust theoretical framework for investigating the behavior of the sensor’s coating. By combining the Young–Laplace equation and Hooke’s law, we gain a comprehensive understanding of how pressure and material properties interact within the sensor. This framework enables quantitative analysis of the sensor’s performance and serves as a basis for optimizing sensor design strategies, ultimately improving the sensor’s accuracy, stability, and reliability.

### 2.3. Physical Simulation Model of the Structure

Finite element simulation, integrated with mechanical theory, is an essential tool for evaluating the reliability and performance of encapsulated sensor structures. In this study, we focus on investigating the influence of encapsulation parameters, particularly the thickness of the parylene coating, on the sensor’s operational performance under various conditions.

**Figure 3:**
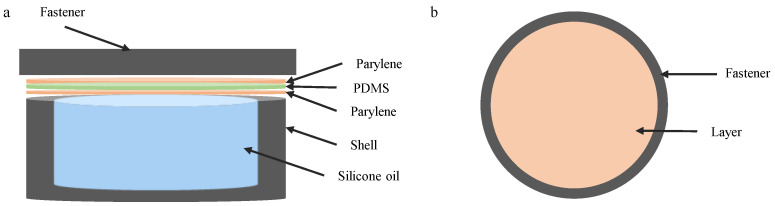
The simulation schematic of the pressure sensor. (**a**) The exploded view of the pressure sensor. (**b**) Top view of the pressure sensor.

As shown in Figure 3, the simulation model includes key components, such as fasteners, the pressure chamber, PDMS, parylene coating, and filling materials. The material properties are assigned based on actual dimensions and specifications, with the outer shell being a modeled ABS copolymer. Parylene N and PDMS are selected as coating materials. Table 1 provides detailed material parameters. The experimental environment is maintained at a constant 37 °C, and a plane stress assumption is applied. Uniform pressure is applied to the coating surface, while thermal coupling effects are excluded to enhance the computational efficiency.

The geometry of the encapsulation structure of the pressure sensor was modeled in three dimensions, simplifying certain structural elements such as through-holes and fasteners to focus on the essential aspects for computational efficiency. The housing is represented as a cylinder with an inner diameter of 5 mm and an outer diameter of 6 mm, above which a coating layer with varying thicknesses is stacked. The space between the coating and the housing is filled with silicone oil. This simplification enables a more focused analysis while maintaining the key features of the encapsulation.

**Figure 4:**
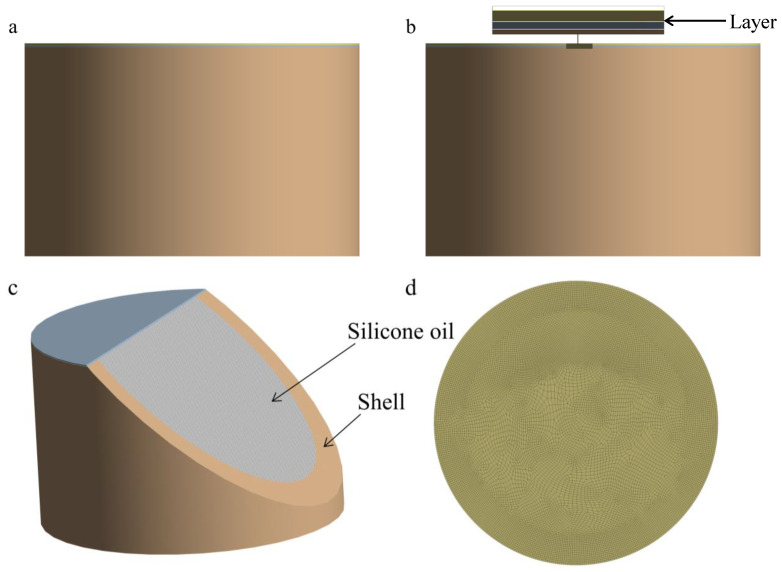
Finite element model of packaging structure. (**a**) Front view of the finite element model. (**b**) Detailed view of finite element model coating structure. (**c**) Detail view of shell and silicone oil for finite element model. (**d**) Mesh diagram of finite element model coating.

The final 3D structure, including its mesh distribution, is depicted in Figure 4. During the meshing process, the model was discretized using tetrahedral elements, specifically high-order SOLID187 elements, which are widely used for their accuracy, especially in handling complex geometries and areas with large stress gradients. The coating structure, with a 12 mm diameter, was meshed using elements sized at 150 µm, ensuring the model’s precision. Given the simplicity of the overall geometry, the model was simulated as a full model rather than employing a half or quarter model, which would have been a common technique for simplifying simulations.

The final model comprises approximately 300,000 tetrahedral elements and 204,560 nodes, which together form a highly detailed and accurate discretized representation of the sensor’s coating structure. To ensure the quality of the mesh, several critical parameters, such as the Jacobian determinant and the maximum and minimum angles, were thoroughly checked. The results confirmed that the mesh adhered to high-quality standards, with no significant distortion or irregularities. This rigorous validation ensures the reliability and stability of the finite element analysis, providing a solid foundation for accurate stress calculations in later stages of the simulation.

### 2.4. Testing Platform

**Figure 5:**
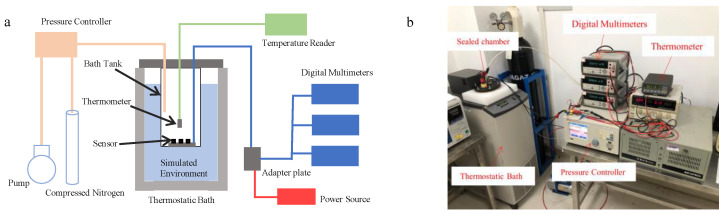
The schematic diagram of testing platform. (**a**) Pressure sensor test platform. (**b**) Pressure sensor experimental test.

In the verification experiment, the encapsulated pressure sensors were placed inside a sealed chamber of the experimental platform (Figure 5). The chamber is connected to a pressure controller (Fluke PPC4) and a thermometer (Fluke 1502A) via two sealed tubes, while the circuit board connecting the sensor is located outside the chamber, with the sensor and circuit board linked by wires. Additionally, the pressure controller (Fluke PPC4) consists of a vacuum pump, compressed nitrogen, and other equipment. The sealed chamber is placed in a high-precision constant temperature water bath (Fluke 7341) to ensure that the sensor operates at a constant temperature. The temperature of the gas inside the chamber is monitored by a thermometer. This experimental platform can simultaneously test three pressure sensors, and the output of the sensors is measured using a digital multimeter (Agilent 34410A, $6\frac{1}{2}$ digit resolution).

## 3. Experiment and Discussion

### 3.1. Finite Element Analysis

In accordance with the practical application scenarios of implantable pressure sensors and the guidelines specified in GB/T 15478-2015 Performance Test Methods for Pressure Sensors, we subjected the sensor’s coating to at least three cycles of pressure loading. To assess the input–output relationship, six evenly distributed pressure points across the full-scale measurement range were selected, encompassing the upper and lower limits of the sensor’s range. The pressure measurement range was set between 100 kPa and 150 kPa, with increments of 10 kPa. At each pressure point, the corresponding output was recorded and repeated at least three times for both upward and downward calibrations to ensure accuracy.

To minimize systemic experimental errors, the pressure variation within the testing system was meticulously controlled to remain within 1 Pa. This strict control helps ensure the reliability and repeatability of the experimental results.

Under normal operating conditions, the surface of the sensor’s coating is directly exposed to the environment. In the static analysis, we applied a pressure starting from 100 kPa to the entire coating surface (including the wall thickness area in contact with the cylindrical casing), which was then increased to 150 kPa. The bottom surface of the model was fixed, and the contact surface between the coating and the cylindrical casing was constrained as a boundary. This setup simulated the actual pressure distribution and mechanical interaction that the sensor experiences.

**Figure 6:**
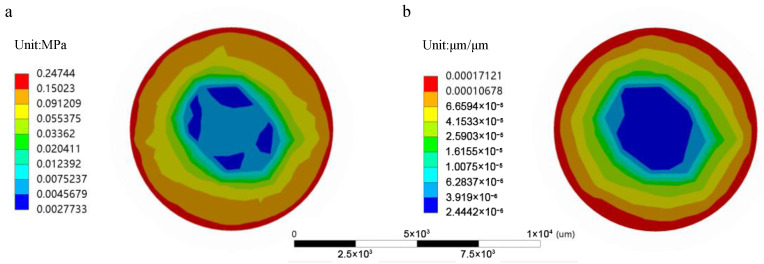
Simulation of the model’s bottom surface. (**a**) Equivalent stress distribution on the bottom surface of the model. (**b**) Equivalent strain distribution on the bottom surface of the model.

**Figure 7:**
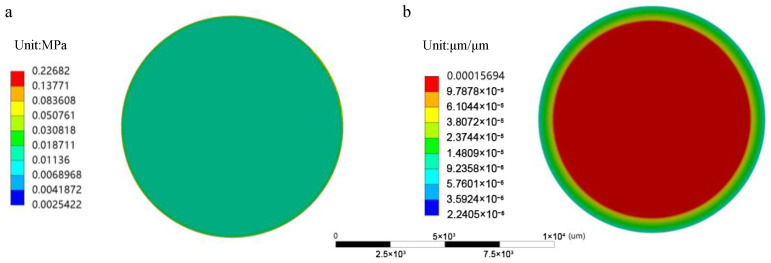
Simulation of the layer’s bottom surface. (**a**) Equivalent stress distribution on the bottom surface of the layer. (**b**) Equivalent strain distribution on the bottom surface of the layer.

Since the pressure sensor is installed at the bottom surface inside a cylindrical cavity, surrounded by silicone oil with an outer coating designed to detect small pressures, we were able to capture the static structural results at the bottom surface of the cylindrical cavity, as shown in Figure 6. The maximum internal stress observed within the structure was 247.44 kPa, located at the edge of the cavity bottom, particularly at the point where the silicone oil contacts the bottom surface of the cylindrical cavity. The maximum strain value was 0.00017121 µm/µm, also concentrated near the same edge region. However, this result did not align with the ideal results derived from the uniform applied stress. Therefore, we also collected the static results from the bottom surface of the coating. As shown in Figure 7, except for a slight increase in stress at the contact area between the coating and the cavity wall thickness, most of the surface showed a uniform stress distribution. This localized increase in stress is a normal phenomenon related to the system’s geometry and boundary conditions. Furthermore, the coating structure transmits pressure through elastic deformation. To avoid pressure losses, we ensured that the coating material had good biocompatibility, flexibility, and chemical stability, thereby improving the measurement accuracy of the pressure sensor.

The results indicate that the uniform external pressure, once transmitted through the coating structure to the silicone oil, caused the silicone oil to compress, leading to the uneven stress distribution, as shown in Figure 6. Interestingly, this behavior deviates from the theoretical incompressibility of silicone oil, suggesting that the observed edge stress concentration may be due to simulation limitations or numerical artifacts rather than actual physical properties. To account for potential system errors, we calculated the average equivalent stress across the entire bottom surface of the cavity and used it as the representative measurement value for the sensor. By calibrating this value using the standard C29 sensor chip, we were able to determine the corresponding output voltage based on the measured stress. This method ensures that the measurement results are robust and reliable, minimizing errors caused by localized stress anomalies.

As is well known, additional encapsulation inevitably leads to some degree of performance loss in sensors, and previous studies often employed fixed encapsulation parameters. However, in order to systematically assess the impact of encapsulation components on pressure sensor performance, we conducted quantitative experiments to study the effects of PDMS and parylene coatings on the performance of pressure sensors, with the aim of optimizing the sensor encapsulation design. To ensure statistical relevance and consistency of the results, five samples were tested for each coating thickness, and the experimental data were derived from the average of these measurements.

**Figure 8:**
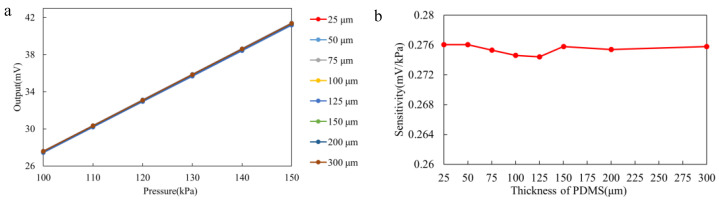
Experimental results for the PDMS simulation group. (**a**) Output curve of the PDMS simulation group. (**b**) Sensitivity change curve of the PDMS simulation group.

In the first set of tests, we investigated the performance of sensors encapsulated with only polydimethylsiloxane (PDMS) coatings. As shown in Figure 8, after encapsulating the sensors with PDMS layers of varying thicknesses, we observed a minimal change in the output sensitivity, which is not significantly affected by the relatively inert nature of the PDMS coatings. This result suggests that the PDMS coating mainly acts as a protective encapsulant and does not have a significant impact on the performance of the sensor. Based on these preliminary results, we proceeded by adding an additional parylene coating to the sensor and continued with further tests.

**Figure 9:**
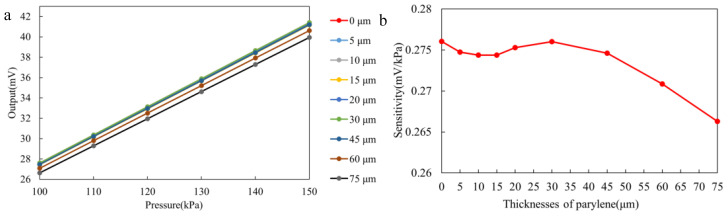
Experimental results for the parylene simulation group. (**a**) Output curve of the parylene simulation group. (**b**) Sensitivity change curve of the parylene simulation group.

In contrast, Figure 9 illustrates a more pronounced effect when the parylene coating is added. The sensor sensitivity decreases as the parylene coating thickness increases. According to Equation (1), this can be explained by the increase in the radius of curvature of the coating as its thickness increases, which reduces the pressure differential between the inside and outside of the coating. This effect leads to a decrease in the mechanical strain on the sensor, which results in a lower sensitivity. These findings are consistent with previous studies on the mechanical interactions between coatings and sensor structures. When the parylene thickness is below 45 µm, the sensor sensitivity changes by a maximum of 0.6014%, which is considered acceptable in gastrointestinal monitoring. However, when the coating thickness reaches 60 µm and 75 µm, the sensitivity drops from 0.2760 to 0.2708 and 0.2662, representing changes of 1.8802% and 3.5321%, respectively. This indicates a significant degradation in the sensor’s performance.

In our experiments, we noted a trade-off between the sensor’s output sensitivity and the encapsulation thickness. The sensor’s sensitivity is generally related to the ratio of small changes in the measurement element relative to the input signal. However, the input signal is constrained by the encapsulation structure. Therefore, improving the sensor’s sensitivity often requires compromising the encapsulation protection structure, and vice versa. This trade-off must be carefully considered and optimized based on the specific requirements of practical applications.

### 3.2. Validation Testing

Based on the results of the simulation analysis, we further validated the specific impact of different parylene coating thicknesses on the performance of the pressure sensor through experiments. Initially, we tested the C29 pressure sensor with only PDMS and a 2 µm parylene composite coating. For ease of testing and analysis, the temperature was set to 37 °C, and the pressure range was set between 100 and 150 kPa. Under these conditions, we were able to measure the sensor’s bias voltage corresponding to the pressure within the chamber.

**Figure 10:**
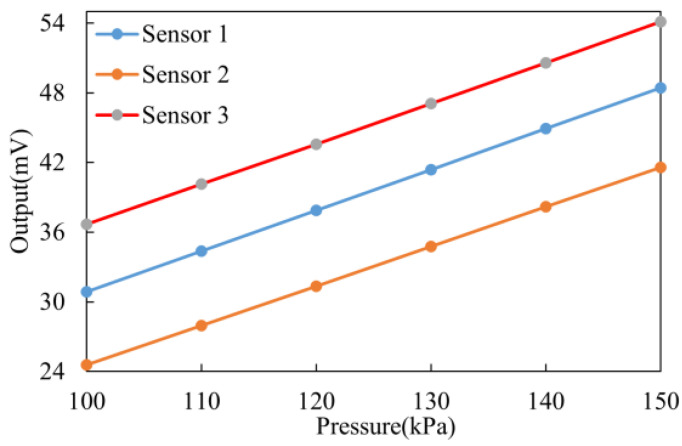
Output curve of the pressure sensors before packaging.

As shown in Figure 10, all three pressure sensors exhibited good linearity within the specified pressure range. To comprehensively and accurately assess the performance of the encapsulated pressure sensor, we utilized sensitivity, non-repeatability error, hysteresis, and maximum non-linearity error as core evaluation metrics. These parameters provide critical insights into the sensor’s accuracy, stability, and overall response under different loading conditions.

(3) $$S=k=\frac{\Delta y}{\Delta x}\times 100\%.$$

where Δy is the output voltage increment, and Δx is the input voltage increment.

(4) $$\xi_{L}=\frac{\max\left|{\bar{y}}_{i}-y_{i}\right|}{Y_{FS}}\times 100\%.$$

where ${\bar{y}}_{i}$ is the mean output of the *i*-th set, is the full-scale output value $Y_{FS}=k\times \left(X_{H}-X_{L}\right)$.

(5) $$\xi_{H}=\frac{\max\left|\Delta {\bar{y}}_{i}\right|}{Y_{FS}}\times 100\%.$$

where $\Delta {\bar{y}}_{i}$ refers to the average difference in forward and reverse outputs for the *i*-th set.

(6) $$E_{NT}=\frac{\max\left|Y_{i}-y_{i}\right|}{Y_{FS}}\times 100\%.$$

where $E_{NT}$ is the maximum non-linearity error, and *Y* is the output on the fitted curve.

To assess the influence of the coating on the performance of the pressure sensor, we conducted a series of experiments under varying pressure levels. The results were analyzed by comparing the sensors’ performance with and without the additional coating layers. This analysis helps determine the optimal coating thickness that balances the sensor’s sensitivity and stability, ensuring reliable pressure measurements across a wide range of applications.

#### 3.2.1. Static Experiment

**Figure 11:**
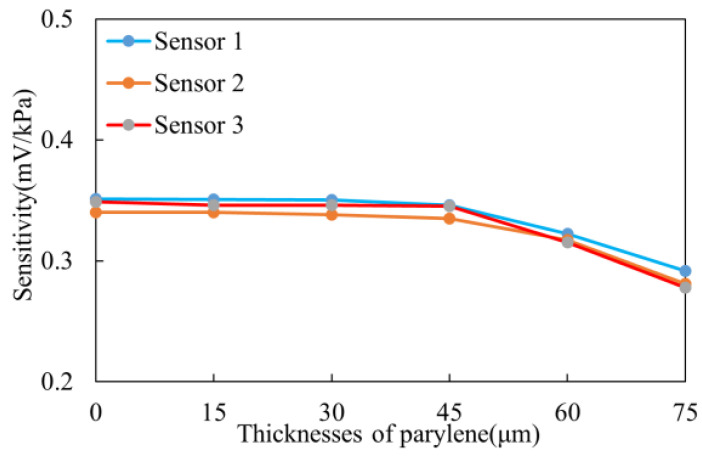
Sensitivity change curve of the experiment group.

Sensitivity and maximum non-linearity error are important technical indicators for pressure sensors, as they directly reflect the accuracy of the sensor. As shown in Figure 11, when the parylene coating thickness is less than 45 µm, the change in sensor sensitivity is minimal, with a sensitivity decrease of no more than 0.45%. However, when the coating thickness exceeds 60 µm, there is a significant reduction in sensor sensitivity, with the maximum change reaching 0.071 mV/kPa. Based on the Laplace pressure model (e.g., (1)), it is important to note that the additional coating reduces the output pressure. Therefore, when the parylene coating thickness exceeds 45 µm, the pressure sensor performance is significantly impacted due to the increased coating thickness. Thus, a proper coating thickness ensures that the sensor is protected from environmental corrosion, while maintaining measurement accuracy.

Table 2 shows the variation in non-linearity error with different parylene coating thicknesses. We observe that as the coating thickness increases, the non-linearity error tends to increase, causing a decline in sensor accuracy. When the parylene coating thickness is reduced to below 30 µm, we observe a significant reduction in non-linearity error variation, with values below 1.0487%. Interestingly, in some cases, the variation in non-linearity error even becomes negative, suggesting that even after coating, the sensor maintains good accuracy. This anomalous behavior is likely due to environmental changes that result in reduced output fluctuations from the pressure sensor. Future studies should further investigate the impact of the experimental environment on sensor performance.

When the parylene coating thickness exceeds 45 µm, sensor accuracy is severely affected. Specifically, when the parylene coating thickness reaches 75 µm, the maximum non-linearity error variation increases to 11.5561%. This indicates that in practical applications, an optimal coating thickness should be chosen based on specific requirements and environmental conditions to ensure measurement accuracy.

**Figure 12:**
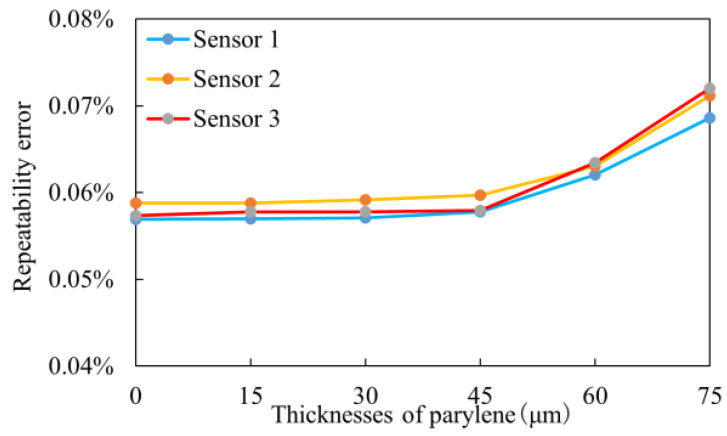
Repeatability error change curve of the experiment group.

Repeatability and hysteresis are also important technical indicators for measuring sensor stability. We conducted repeatability and forward/reverse stroke experiments on three sensors. Figure 12 illustrates the variation in repeatability errors with different parylene coating thicknesses.

As seen in Figure 12, when the parylene coating thickness is less than 45 µm, the repeatability error remains stable, with a maximum variation of no more than 0.7847%. This indicates that, within this thickness range, the coating has a minimal impact on the strain capability and stability of the sensor. However, increasing the thickness from 45 µm to 60 µm causes a significant variation in repeatability errors. This is because, within this range, the coating significantly affects the strain capability of the sensor, leading to performance fluctuations under external forces. When the parylene thickness reaches 75 µm, the repeatability error increases significantly to over 20.4443%, further confirming that thicker coatings reduce the sensor’s strain capability and stability.

Table 3 provides the variation rate of hysteresis errors corresponding to different parylene coating thicknesses. We observe that hysteresis errors increase with increasing coating thickness. When the coating thickness is below 30 µm, the hysteresis error variation does not exceed 0.1273%, indicating good stability. However, when the parylene coating thickness exceeds 45 µm, most sensors show an increasing trend in hysteresis errors. Notably, Sensor 3’s hysteresis error variation rate remained relatively stable, and its performance was even superior. We speculate that this may be related to the impact of temperature variations within the sealed chamber on the pressure sensor. Temperature changes could cause pressure fluctuations inside the chamber, and Sensor 3 may be more sensitive to these changes. It is possible that Sensor 3 employs a special encapsulation process or materials that allow it to better adapt to pressure changes caused by temperature fluctuations, thus maintaining a relatively stable hysteresis error.

Mechanistically, this may be related to the thermal expansion coefficient, elastic modulus, and interface effects between the coating layer and sensor core. As the coating thickness increases, these factors may change, preventing the sensor from quickly returning to its original state after being subjected to stress, thus generating hysteresis errors. However, Sensor 3, due to its specialized packaging, may have been optimized in these areas, reducing the occurrence of hysteresis errors.

Given the instability and unreliability of individual metrics, we considered all the indicators and applied the coefficient of variation method for comprehensive evaluation. The specific steps are as follows:

The experiment is divided into six groups, and the values of each indicator $x_{ij}$ are calculated according to the formula. Here, *i* represents the experimental group, and *j* represents the indicator. Considering that non-linearity errors, hysteresis, and reproducibility errors may negatively affect the results, the indicator values need to be normalized. Due to the differing dimensions of these indicators, normalization is required before aggregation. Based on their negative correlation, the normalization formula is given by (7).

(7) $$x_{ij}^{n}=\frac{x_{ij}-\min x_{ij}}{\max x_{ij}-\min x_{ij}}$$

The effect of sensitivity is positively correlated with the outcome; thus, the normalization formula is given by (8).

(8) $$x_{ij}^{n}=\frac{\max x_{ij}-x_{ij}}{\max x_{ij}-\min x_{ij}}$$

where $x_{ij}^{n}$ is the value of the *j*-th indicator for the *n*-th sensor in the *i*-th experimental group.

The larger the variance of each indicator, the more it reflects the differences between evaluation units. Therefore, in the comprehensive scoring process, a higher weight should be assigned to indicators with larger variances. Based on this principle, the coefficient of variation method typically assigns the weight of each indicator as the ratio of the standard deviation to the mean value of each group, as shown in (9). The normalization formula is given by (10).

(9) $$\omega_{j}=\frac{std(x_{ij}^{n})}{mean(x_{ij}^{n})}$$

(10) $$\omega_{j}^{n}=\frac{\omega_{j}^{n}}{\sum_{j=1}^{4}\omega_{j}^{n}}$$

Finally, the normalized indicator values are multiplied by their respective normalized weights to obtain the comprehensive scores for each layer. The sum of these four scores results in the final comprehensive score, as shown in (11).

(11) $$v_{i}^{n}=\sum_{j=1}^{4}\omega_{j}^{n}x_{ij}^{n}$$

Table 4 presents the variation rates of the comprehensive scores for sensors with different coating thicknesses. When the parylene coating thickness is below 30 µm, the variation rate of the comprehensive score does not exceed 1.0561%, and the maximum score is 0.003. The sensors with this coating thickness provide more accurate and stable pressure measurements. However, when the coating thickness exceeds 45 µm, the comprehensive score increases significantly, and sensor performance is severely affected. Therefore, in specific implantable system applications, it is recommended to avoid using parylene coating thicknesses above 30 µm.

Through a comprehensive analysis of the experimental data, we found that when the parylene coating thickness is maintained below 45 µm, the pressure sensor exhibits good stability across various performance metrics. Specifically, the sensitivity of the sensor decreases by no more than 0.45%, likely due to the minimal effect of a thinner coating layer on the stress transfer and sensor core. Additionally, the variation in the non-linearity error remains below 1.0487%, and the sensor maintains good accuracy even after packaging. Furthermore, the increase in repeatability error does not exceed 0.7847%, indicating good output stability after multiple measurements. The hysteresis error variation is minimal, never exceeding 0.1273%, demonstrating the sensor’s strong recovery capability after force application.

Through comparative analysis, it can be concluded that the parylene encapsulation thickness has a certain impact on the pressure sensor’s performance. Within the pressure range of interest, as the encapsulation thickness increases, a decrease in sensitivity is observed, and non-linear errors, reproducibility errors, and hysteresis tend to increase. Additionally, the experiments and tests were conducted in an environment subject to some degree of interference. While the experimental environment was relatively ideal, it was not under absolute constant temperature and humidity conditions. Temperature fluctuated dynamically within a range of ± 0.1 °C, and relative humidity fluctuated between 40% and 80%. Despite these environmental disturbances, the sensor’s non-linear errors, reproducibility errors, and hysteresis showed a decrease, and the sensor’s performance remained stable.

#### 3.2.2. Dynamic Experiment

**Figure 13:**
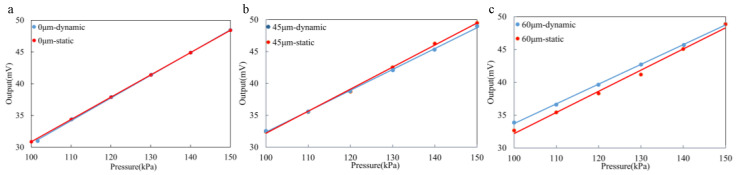
The dynamic output curve of the sensor.(**a**) Comparison of sensor output curves for 0 µm. (**b**) Comparison of sensor output curves for 45 µm. (**c**) Comparison of sensor output curves for 60 µm.

Considering that the pressure within the human gastrointestinal tract is dynamic and fluctuating, and that an additional encapsulation layer inevitably alters the dynamic response and sensitivity of the sensor, we also tested the dynamic response capabilities of the sensor. The rapid inflation method was used to test the dynamic response characteristics of the encapsulated sensor. On the experimental platform, we dynamically changed the pressure within the sealed chamber and recorded the corresponding sensor output data. Figure 13 illustrates the output curve of the sensor.

Testing was conducted with varying parylene coating thicknesses to investigate their effects on sensor performance. When the parylene coating thickness was 0 µm, the dynamic test results remained stable, likely because the sensor was directly exposed to the test environment without the influence of the additional encapsulation layer. This served as the baseline for comparing the performance of the sensor with and without the coating. However, as the coating thickness increased, the dynamic response characteristics of the sensor began to change. When the parylene coating thickness reached 45 µm, the dynamic test results showed a decrease, likely due to the additional stress and thermal insulation effects introduced by the encapsulation layer, which impacted the sensor’s sensitivity and response speed. This thickness represents a critical point where the encapsulation layer starts to have a notable effect on sensor performance, as it begins to alter both the mechanical and thermal properties of the sensor.

As the coating thickness increased further to 60 µm, the dynamic test results exhibited significant anomalies. This could be attributed to the excessive thickness of the encapsulation layer, which caused an uneven internal stress distribution within the sensor and deteriorated heat conduction performance, leading to instability in the sensor’s response to rapid pressure changes. The thicker encapsulation layer likely obstructed the sensor’s ability to react quickly to pressure changes, which is critical in dynamic environments. More critically, when the coating thickness reached 75 µm, there were periods when effective output signals could not be obtained. An overly thick coating layer likely restricted the rapid response ability of the sensor core, and the faster rate of pressure application exacerbated this limitation, preventing the sensor from capturing and outputting pressure change information in a timely and accurate manner.

This study indicates that when the parylene coating thickness is below 45 µm, the dynamic data output of the sensor is consistent with the static data, and the response time is short. This makes the sensor suitable for high-precision, high-sensitivity applications, such as biological signal monitoring. This behavior is likely due to the minimal impact of the coating layer within this thickness range, which allows the sensor to maintain its original sensitivity and response speed. Beyond this thickness, the coating introduces detrimental effects on the sensor’s dynamic behavior, underlining the importance of optimizing the coating thickness to balance protective benefits with dynamic performance requirements.

## 4. Conclusions

In this study, we introduced an innovative repackaging technique featuring a multi-layer structure consisting of silicone oil, PDMS, and parylene. Our research primarily focused on evaluating the performance of a pressure sensor encapsulated with parylene and PDMS coatings. To understand the impact of coating thickness on sensor behavior, we employed both finite element simulations and experimental validation. The results highlighted the crucial role of encapsulation layers, especially the parylene coating, in influencing the sensor’s mechanical response, including its sensitivity, linearity, and stability under both static and dynamic conditions.

Our simulations revealed that the effect of PDMS thickness, within 25–300 µm, could be ignored with regard to the measurement accuracy. When the PDMS thickness is constant, the internal stress distribution within the sensor became more uneven as the parylene coating thickness increased, leading to a decrease in sensitivity and an increase in non-linear errors. This effect was particularly pronounced when the coating thickness exceeded 45 µm. Experimental results supported these findings, showing that the sensor’s dynamic response remained most stable when the parylene coating thickness was below 45 µm. Beyond this threshold, the sensor exhibited significant performance degradation, including reduced sensitivity, increased non-linearity, and unstable dynamic responses, particularly when the coating thickness reached 75 µm.

Additionally, this study lays a solid foundation for the development of gastrointestinal monitoring systems and contributes to advancing diagnostic and therapeutic technologies for gastrointestinal diseases. However, while the current sensor performance is promising, there remains significant potential for further optimization. In our future research, we aim to deepen our understanding of the encapsulation mechanisms and investigate how various encapsulation parameters, such as coating thickness, material interaction, and structural design, influence sensor performance. Optimizing these design parameters by understanding the specific requirements of the application and conducting thorough testing will help us fine-tune the design to achieve a better balance between biocompatibility, mechanical strength, and sensor stability. Furthermore, further investigations are required to assess its long-term reliability and stability under clinical conditions. This will involve assessing the sensor’s biocompatibility, resistance to environmental factors such as corrosion, and overall performance over extended periods. By conducting these studies, we aim to provide a more thorough understanding of the sensor’s durability and its potential for practical applications in medical monitoring systems.

## Figures and Tables

**Table 1:** Data parameters of materials.

Materials	Young’s Modulus (MPa)	Poisson’s Ratio	Density (g/cm^3^)
ABS	2000	0.394	1.05–1.18
PDMS	2100–2400	0.45–0.50	0.95–1.10
Parylene N	2400	0.40	1.10–1.12

**Table 2:** The rate of non-linear error change in the experimental group.

Thickness (µm)	Sensor 1	Sensor 2	Sensor 3
15	0.0768	−0.3400	−0.1730
30	1.0487	0.7122	−0.6942
45	26.7157	1.8213	−0.5037
60	52.7542	15.9491	3.2175
75	97.7247	40.2978	11.5561

**Table 3:** The rate of hysteresis error change in the experimental group.

Thickness (µm)	Sensor 1	Sensor 2	Sensor 3
15	−0.0797	−0.1895	0.1273
30	−0.2723	−0.3062	−0.1800
45	7.8066	−0.7024	0.1133
60	12.1647	−0.0012	−0.3816
75	19.1249	0.0023	0

**Table 4:** The rate of overall score change in the experimental group.

Thickness (µm)	Sensor 1	Sensor 2	Sensor 3
15	0.02884	−0.3463	0.9741
30	0.1392	1.0561	0.4453
45	14.0392	3.5939	1.0533
60	28.9549	22.8411	15.3181
75	51.5562	58.3080	36.5723

## Data Availability

The original contributions presented in this study are included in this article. Further inquiries can be directed to the corresponding author.
